# Aortic Valve Papillary Fibroelastoma Associated with Acute Cerebral Infarction: A Case Report

**DOI:** 10.1155/2013/485029

**Published:** 2013-01-10

**Authors:** Nobuhiro Takeuchi, Masanori Takada, Koichi Fujita, Yoshiharu Nishibori, Takao Maruyama, Kazuyoshi Naba

**Affiliations:** ^1^Department of Cardiology, Kawasaki Hospital, Kobe, Japan; ^2^Department of Laboratory Medicine, Kawasaki Hospital, Kobe, Japan

## Abstract

An 80-year-old woman with a history of congestive heart failure, atrial fibrillation, and hypertension was transferred to our institution with hematemesis. Her drug regimen included 2 mg warfarin potassium/day to prevent thromboembolic events. Transthoracic echocardiography (TTE) performed at 78 years of age revealed a mass attached to the noncoronary cusp and a cardiac tumor was suspected. The patient declined surgery and was meticulously followed up with periodic TTE. Upper gastroendoscopy revealed a gastric ulcer with an exposed blood vessel; anticoagulant therapy was ceased. On day 15 of admission, acute cerebral infarction occurred. Heparin sodium and warfarin potassium were administered rapidly, and her symptoms improved. TTE revealed no alteration of the mobile, string-like mass attached to the noncoronary cusp. Cardiac tumor was considered the cause of cerebral infarction, and the patient consented to surgical therapy. Pathological examination of the resected tumor suggested papillary fibroelastoma (PFE). Although no guidelines exist for PFE management, a mobile, cardiac tumor necessitates surgical resection to prevent thromboembolic events, even when small in size.

## 1. Introduction

Primary cardiac tumors are rare, accounting for 0.002%–0.3% of all cardiac tumors [1]; moreover, 70% of these tumors are benign [1]. Sometimes, primary cardiac tumors can cause thrombosis including cerebral or myocardial infarction. Here we report the case of a female patient with an aortic valve tumor that was discovered during heart failure therapy; she experienced that was cerebral infarction after ceasing anticoagulant therapy due to gastric ulcer. The aortic valve tumor was successfully resected and a papillary fibroelastoma (PFE) of the aortic valve was diagnosed on the basis of the pathological findings.

## 2. Case Presentation

**Figure 1:**
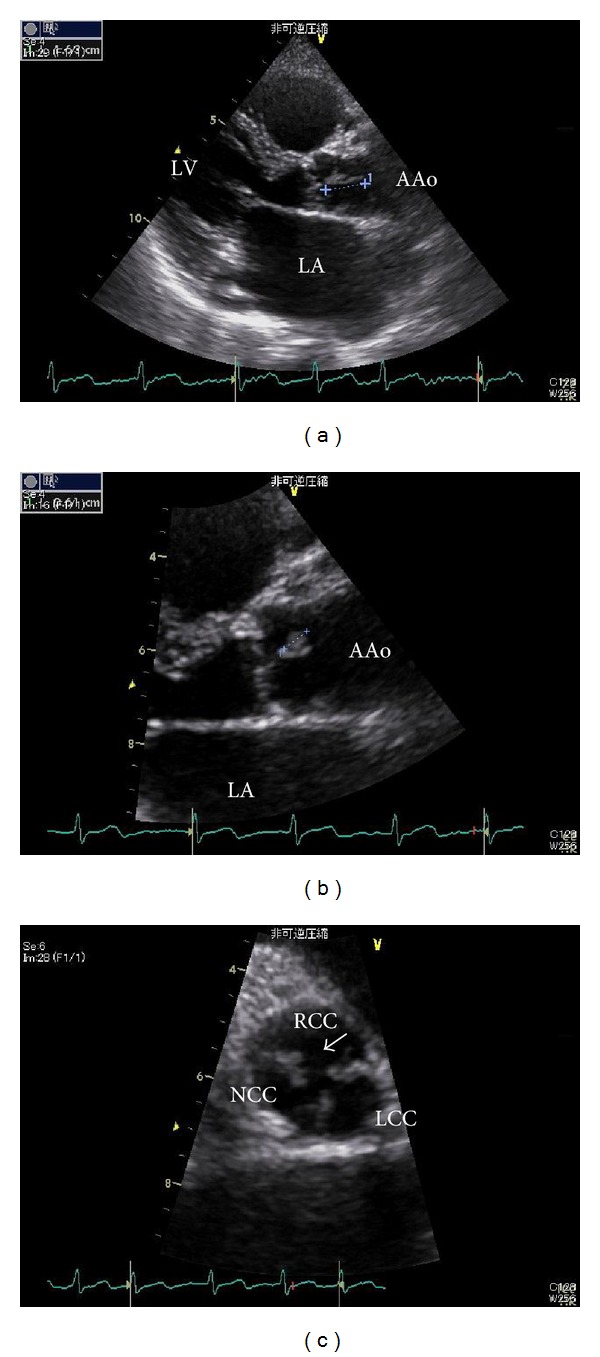
Transthoracic echocardiography. Transthoracic echocardiography revealed a mobile, string-like mass 7 mm in size attached to the noncoronary cusp. (a) and (b) left parasternal short axis views, (c) left parasternal short axis view at the level of aortic valve.

An 80-year-old woman was transferred to our institution with hematemesis. She had a history of congestive heart failure, atrial fibrillation, and hypertension; moreover, she had a history of hepatocellular carcinoma at the segment S5 caused by chronic hepatitis C, which was treated by transcatheter embolism. She underwent treatment for congestive heart failure and was prescribed warfarin potassium at a dose of 2 mg/day to prevent thromboembolic events. Transthoracic echocardiography (TTE) performed at 78 years of age revealed a 7 mm small, mobile, string-like mass attached to the noncoronary cusp (Figure 1). Infective endocarditis was excluded on the basis of the following findings: blood chemical analyses revealed no elevation of inflammation markers, blood cultures were negative for microorganisms, and the patient's body temperature was within the normal range. A cardiac tumor, including metastasis of the hepatocellular carcinoma, was suspected, and surgical treatment was recommended to prevent thromboembolic events. However, the patient declined surgery; thus, she was carefully followed up with periodic TTE.

Thereafter, the patient was transferred to our institution with complaints of upper abdominal pain and hematemesis. Her blood pressure was 130/90 mmHg, and heart rate was 130 beats/min. Physical examination revealed mild anemia, a soft, flat abdomen with no palpable masses, and normal bowel sounds. On auscultation, her chest was clear and no abnormal murmurs could be heard. Chest radiography did not present any signs of cardiomegaly or pulmonary congestion. Electrocardiography revealed atrial fibrillation. Blood chemical analyses revealed mild anemia (red blood cell count: 255 × 10^4^/*μ*L; hemoglobin levels: 8.2 g/dL), slightly low platelet count (12.5 × 10^4^/*μ*L), slightly elevated serum urea nitrogen levels (29.6 mg/dL), mild hypoproteinemia (6.1 g/dL), mild hypoalbuminemia (2.3 g/dL), slightly elevated C-reactive protein levels (1.3 mg/dL), and coagulation dysfunction (prothrombin time, 49%; fibrin/fibrinogen degradation products, 27.9 *μ*g/mL; and D-dimer, 14.7 *μ*g/mL). Upper gastroendoscopy revealed a gastric ulcer with an exposed blood vessel situated posteriorly in the body of the stomach. Anticoagulant therapy was ceased, and a proton pump inhibitor was administered.

**Figure 2:**
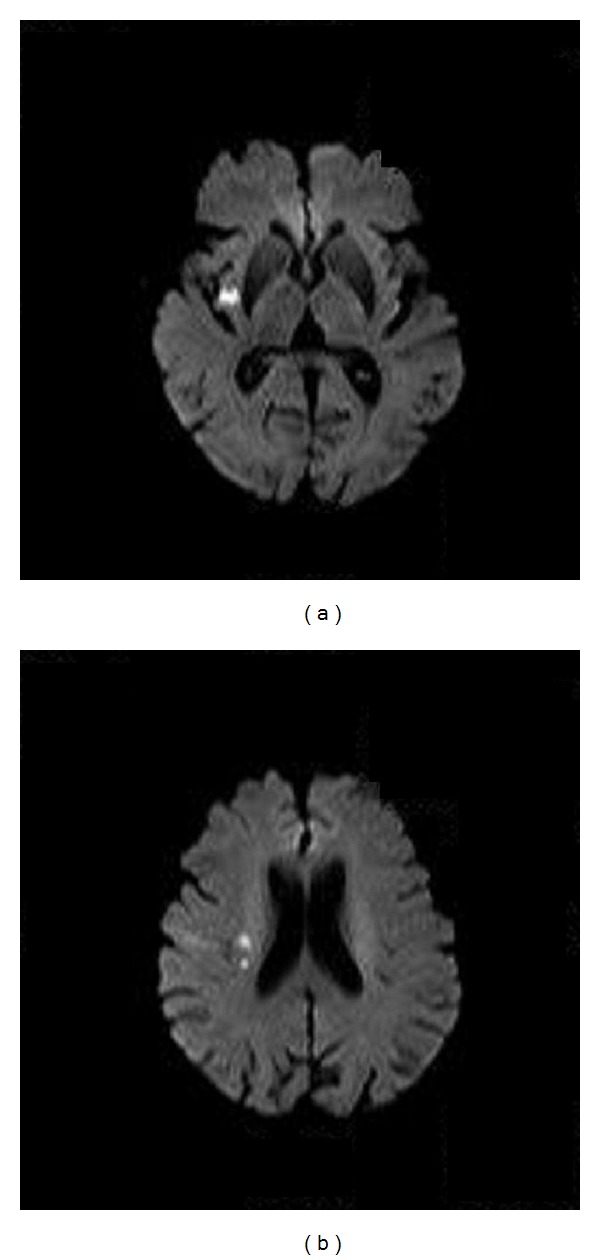
Magnetic resonance imaging of the brain.High-intensity signal lesions in the right insular cortex (a), and right corona radiata (b).

On day 15 of admission, the patient experienced mild impairment of consciousness, disturbance of speech articulation, and left facial paralysis. Magnetic resonance imaging of the brain revealed high-intensity signal lesions in the right insular cortex and right corona radiate (Figure 2). Acute cerebral infarction was diagnosed and heparin sodium and warfarin potassium were administered; subsequently her symptoms improved. TTE revealed no alteration of mobile, string-like mass attached to the noncoronary cusp. Moreover, moderate mitral and mild aortic regurgitations were evident, although ejection fraction was unaffected. We considered the tumor to be the cause of acute cerebral infarction, although the possibility of atrial fibrillation causing the infarction could not be excluded. The patient consented to surgical therapy, and surgery was performed 1.5 months after the cerebral infarction occurred. Cardiopulmonary bypass was established and the ascending aorta was opened by median sternotomy. The tumor revealed attachments to the noncoronary, left, and right coronary cusps; eventually tumor resection was performed. Furthermore, after resection, the maze procedure was performed for atrial fibrillation.

**Figure 3:**
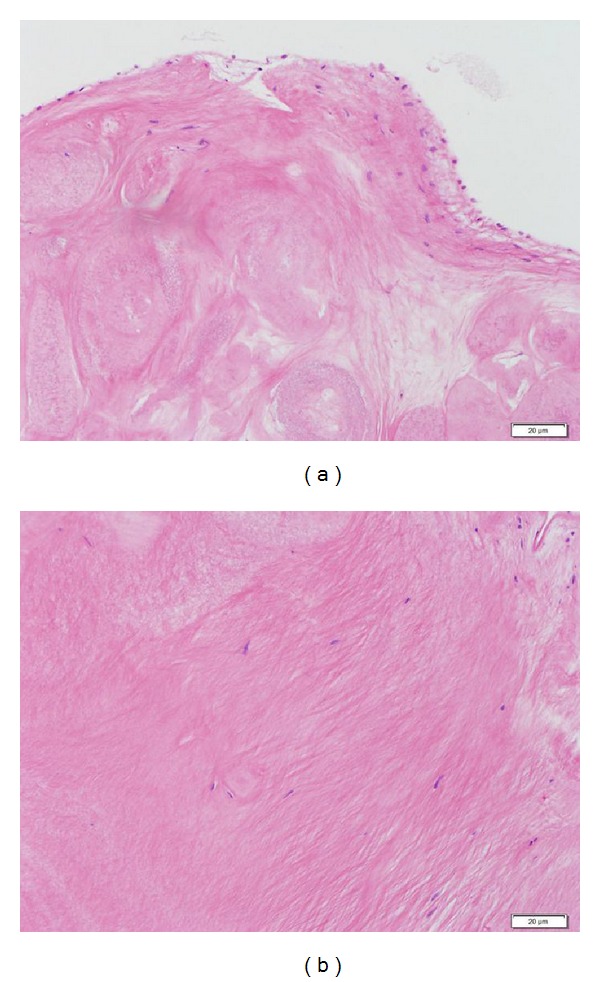
Microscopical analysis of the resected specimen revealed a single squamous cell layer covering the surface (a), and a core comprising aggregated elastic fibers and hyaline material (b).

Pathological examination of the resected specimen revealed that it is comprised of a core of aggregated elastic fibers and hyaline material covered with a single squamous cell layer (Figure 3). There was no evidence of metastatic hepatocellular carcinoma; however, the pathologic findings were consistent with PFE. Atrial fibrillation was restored to sinus rhythm, and the postoperative course was uneventful.

## 3. Discussion

Primary cardiac tumors are extremely rare and 70% of them are usually benign [1]. PFE accounts for 8% of primary cardiac tumors and is the third most common tumor, after lipoma and myxoma [2]. Moreover, PFE can develop at any location of the endocardium; however, 84% of PFE lesions are valvular, (most commonly developing in the aortic valve, followed by the mitral valve) [3]. In some cases [4, 5], PFE was discovered after the occurrence of thrombosis such as cerebral or myocardial infarction. In others [6, 7], PFE was observed incidentally during surgery or autopsy. With recent advances in echocardiography, PFE has even been detected at routine health checkups or during followups for other diseases [8].

Generally, PFEs are about 1 cm in diameter and are usually difficult to detect on TTE. In fact, PFE presents symptoms pertaining to thrombosis alone, if any. In cases of repeated cerebral infarction, PFE is often detected incidentally on transesophageal echocardiography; however, detection on TTE is infrequent. Rarely, aortic valve PFE causes sudden death by obstructing the left ventricular outlet tract or coronary artery ostia. Therefore, aortic masses require careful diagnosis and management. PFE has no characteristic echocardiographic findings, and diagnosis is usually confirmed by pathological examination of the resected specimens. PFE has many white papillary fragments, with frond-like projections reminiscent of a sea anemone, and adheres to the endocardium. Histologically, PFE comprises hyperplastic endothelial cells, with a hard, central connective tissue core surrounded by loose connective tissue. The connective tissue core adheres to the connective tissue of the endocardium and comprises rich elastic fibers. The loose connective tissue comprises mucopolysaccharide, smooth muscle cells, and hyperplastic (but not atypical) endothelial cells.

When presentations suggestive of PFE are revealed by echocardiography, heparin sodium administration should be initiated, and tumor resection should be considered as soon as possible [3]. Because of its fragility, PFE itself sometimes causes thrombosis. Frequently, the thrombus formed on the PFE surface itself causes thrombosis [9]. Although no guidelines on PFE management exist, the key issue is whether PFE should be treated surgically or conservatively with anticoagulant therapy. Ngaage et al. [10] suggested that asymptomatic patients with mobile masses >1 cm in the left heart chambers should be treated surgically to prevent thromboembolic events or sudden death. Conversely, Sun et al. [11] suggested that asymptomatic patients with small, immobile masses in the left heart chambers should be carefully observed by echocardiography until symptoms emerge or tumors become large and mobile. However, surgery is recommended in all symptomatic patients, because of the increased risk of thromboembolic events and sudden death. In our case, cerebral infarction occurred during the observation period for cardiac tumor. To some extent, although atrial fibrillation might have contributed to cerebral infarction, the patient elected to undergo tumor resection after the improvement of her gastric ulcer, with concerns that recurrent cerebral infarction would be devastating.

PFE is considered a hamartoma or organized thrombus. Therefore, complete resection of the lesion and papillary proliferations is considered sufficient to prevent tumor recurrence. In many cases [12], tumor resection is performed alone, without valve repair or replacement with a prosthetic valve. Conversely, valve replacement is performed when complete tumor resection is difficult because the tumor is acaulescent or widely attached to valves. Gowda et al. [13] analyzed 725 cases of PFE and reported that average patient age at diagnosis was 60 years with 55% of patients being males. Moreover, PFE was located in the aortic valve in 44%, mitral valve in 35%, and tricuspid valve in 15% of the patients. Furthermore, the following clinical symptoms of PFE were reported: transient ischemic attack or cerebral infarction (17%), angina pectoris (14%), myocardial infarction (4%), and sudden death (3%). Of the 425 surgically treated patients, 81% were treated with simple tumor resection, 9% with tumor resection and valve repair, and 10% with prosthetic valve replacement.

Endocarditis should be ruled out whenever we encounter a mass in a cardiac valve. Usually, when fever is absent and blood chemistry analyses reveal no elevation of inflammatory markers, endocarditis is unlikely. When inflammation with fever is evident, the diagnosis is more complicated. In our case, the patient exhibited no fever during the followup of cardiac mass, and thus endocarditis was considered unlikely.

## 4. Conclusion

Upon the discovery of a cardiac tumor, it is important to rule out endocarditis. Especially when the tumor is mobile, even when small in size, operative resection should be considered as soon as possible to prevent devastating thromboembolic events.
